# Real-time change in dynamic cerebral autoregulation after acupuncture at GB34 (Yanglingquan): a self-controlled study

**DOI:** 10.3389/fphys.2026.1681930

**Published:** 2026-02-02

**Authors:** Weijun Zhang, Lihua Wu, Ziyu Ye, Min Wan, Wen Fu, Baile Ning, Shanze Wang, Li Xiong, Jia Liu, Pandeng Zhang, Jingxin Zhong, Wenbin Fu

**Affiliations:** 1 Department of Brain Function, The Second Affiliated Hospital of Guangzhou University of Chinese Medicine, Guangzhou, China; 2 Brain Function Examination Center, The Affiliated Brain Hospital, Guangzhou Medical University, Guangzhou, China; 3 Rehabilitation Medicine College, Henan University of Chinese Medicine, Zhengzhou, China; 4 Clinical Medical College of Acupuncture Moxibustion and Rehabilitation, Guangzhou University of Chinese Medicine, Guangzhou, China; 5 The Second Clinical Medical College, Guangzhou University of Chinese Medicine, Guangzhou, China; 6 Department of Rheumatology, The First Affiliated Hospital of Henan University of Chinese Medicine, Zhengzhou, China; 7 Department of Acupuncture and Moxibustion, The Second Affiliated Hospital of Guangzhou University of Chinese Medicine, Guangzhou, China; 8 Clinical Trials Centre, The Eighth Affiliated Hospital of Sun Yat-sen University, Shenzhen, China; 9 Institute of Advanced Computing and Digital Engineering, Shenzhen Institutes of Advanced Technology, Chinese Academy of Sciences, Shenzhen, China

**Keywords:** acupuncture, cerebral blood flow, cerebrovascular function, dynamic cerebral autoregulation, transcranial doppler

## Abstract

**Background:**

Few interventions have been proven to improve dynamic cerebral autoregulation (dCA). GB34 is a common acupoint for motor function treatment in the clinic. However, the effects of acupuncture on dCA have never been reported and whether acupuncture at GB34 can improve dCA is unknown.

**Methods:**

A self-controlled interventional study was conducted. Each participant randomly received two acupuncture interventions, including active acupuncture and sham acupuncture. Transcranial Doppler and servo-controlled finger photoplethysmography were used to record continuous cerebral blood flow velocity in the bilateral middle cerebral arteries and continuous blood pressure to assess dCA with transfer function analysis during acupuncture intervention.

**Results:**

Twenty healthy volunteers were enrolled. The left and right phase values in the low-frequency band after acupuncture treatment were significantly higher than baseline levels. Meanwhile, a reduction in heart rate was observed after acupuncture treatment. In contrast, no real-time changes were observed in any parameters after sham acupuncture intervention.

**Conclusion:**

The results suggested that treatment with acupuncture at GB34 increased dCA in young healthy volunteers. The increased dCA may contribute to the beneficial effects of acupuncture on cerebrovascular function.

**Clinical Trial Registration:**

https://www.chictr.org.cn/showproj.html?proj=121012, identifier ChiCTR2100042762.

## Background

Cerebral autoregulation (CA) is the ability of the brain to control changes in cerebral blood flow (CBF) in response to changes in arterial blood pressure (BP) (Panerai et al., 2022). Because the advent of transcranial Doppler (TCD) allows much higher temporal resolution for measurements of CBF, dynamic cerebral autoregulation (dCA), which refers to the transient response of CBF to rapid changes in BP across seconds or minutes, has gradually become more popular in clinical studies (Claassen et al., 2021). Currently, dCA is known to be compromised in various medical conditions, such as stroke (Xiong et al., 2017), subarachnoid hemorrhage (Otite et al., 2014), Parkinson’s disease (Chen et al., 2022), diabetes mellitus (Vianna et al., 2015), panic disorder (Wang et al., 2010), and depression (Zhang et al., 2022a). With the development of dCA, improving impaired dCA has become a focus. However, few interventions have been proven to improve CA function.

Acupuncture has been developed for thousands of years as a traditional treasure in China. As a unique therapy of Chinese medicine, acupuncture is popular because of its exact clinical effect and few adverse side effects. However, the underlying brain function mechanisms of acupuncture are not fully understood, especially given the lack of dynamic, continuous, visual and objective evidence. Compared with neuroimaging techniques, TCD can dynamically monitor CBF velocity (CBFV) to assess changes in CBF. Used in combination with the continuous arterial BP device, TCD allows changes in dCA to be assessed. Therefore, it is feasible to observe the changes in dCA during acupuncture.

GB34, called Yanglingquan, is a common acupoint for motor function treatment in the clinic. It is frequently chosen for neurological diseases, such as stroke (Wang et al., 2022; Ning et al., 2017) and Parkinson’s disease (Yeo et al., 2014). By increasing CO_2_ reactivity specifically in the ipsilateral middle cerebral artery (MCA), GB34 acupuncture treatment improves the vasodilatory potential of the cerebral vasculature to compensate for fluctuations caused by changes in external conditions (Moon et al., 2019). However, whether acupuncture at GB34 can also improve dCA function in humans is unknown.

Since both cerebral vasomotor reactivity and CA are the main mechanisms of cerebral blood flow regulation (Liu et al., 2022), in the present study, we hypothesize that in the same subject, active acupuncture at GB34 improves dCA, while sham acupuncture does not.

## Methods

### Participants

This prospective study was approved by the ethics committee of the Second Affiliated Hospital of Guangzhou University of Chinese Medicine (BF2020-186-05). All subjects gave their informed consent prior to their inclusion in the study. This trial is registered at the Chinese Clinical Trial Registry (ChiCTR2100042762). Twenty healthy volunteers aged 20 to 30 were enrolled in this study from September 2021 to December 2021. No participants were experiencing or had a history of physical or mental diseases, including cerebrovascular disease, traumatic brain injury, hypertension, frequent arrhythmias, diabetes mellitus, hyperthyroidism, generalized anxiety disorder, depression, and insomnia. Participants with regular alcohol or nicotine consumption, poor cooperation or poor temporal bone windows were excluded from the study.

### Acupuncture interventions

**FIGURE 1:**
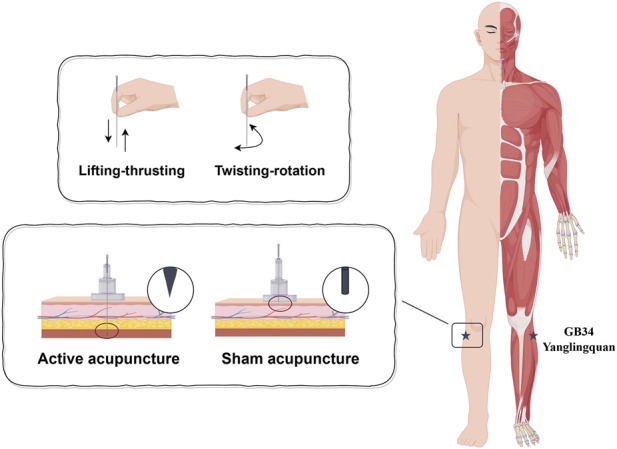
Acupuncture interventions.

**FIGURE 2:**
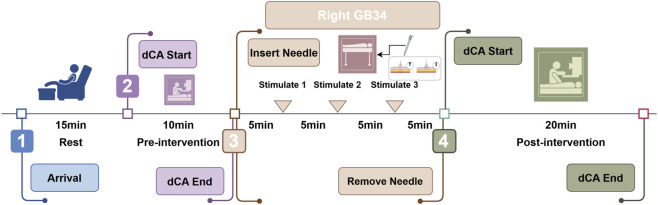
Timeline of the study design.

Each participant randomly received two acupuncture interventions. One of them was active acupuncture, and the other was sham acupuncture. All acupuncture treatment procedures were performed by the same experienced acupuncturist. The selected acupoint, the right GB34, is located on the fibular aspect of the right leg in the depression anterior and distal to the head of the fibula (Figure 1). Acupuncture was performed using a sterile, single-use tube needle (Hwato Suzhou Medical Instruments, Suzhou, China). As shown in Figure 1, the active acupuncture involved using a stainless steel acupuncture needle that was vertically inserted through the skin to a depth of approximately 20 mm, while the sham acupuncture involved using a blunt-tipped needle that was vertically inserted but unable to pass through the skin. The stimulation was applied by rotation of the needle at approximately 1.5 Hz until the participant experienced Deqi sensation (the needle reaction) or reached the upper limit of 20 s for the stimulation. The needle was twisted once every 5 min, with removal of the needle after 20 min (Figure 2).

### Randomization and blinding

Independent researchers generated random numbers using SPSS statistical software 18.0 (IBM Corp., Armonk, NY, United States) and concealed the random sequence via the envelope method. This sequence determined the order of stimulus conditions (active acupuncture, sham acupuncture) administered to participants. The participants were blinded to the stimulus conditions through the use of the plastic sleeve acupuncture device.

### Sample size

The current study is a cross-over investigation, which there is no prior information to base a sample size on. According to the rule of thumb for a pilot study, a minimum sample size of 12 was recommended (Julious, 2005). Referring to the sample size of a previous study about effects of GB34 acupuncture on cerebral vasomotor reactivity (Moon et al., 2019), the estimated sample size of our study was 20, which is a common sample size in physiological crossover experiments.

### Study design

The researchers screened healthy subjects. If the volunteer met the inclusion criteria, a dedicated researcher introduced the study to the volunteer. Once written informed consent was obtained, subjects were randomly received two acupuncture interventions. The interval between two acupuncture interventions was at least 1 week. Before and after each acupuncture interventions, phase and gain were used to quantify the dCA (Figure 2).

Primary outcome measures were the left and right phase levels after GB34 acupuncture treatment. Secondary outcome measures were other CA parameters, MCA velocity, Mean BP, Heart rate after GB34 acupuncture treatment. In addition, subject baseline data, such as demographic characteristics, were collected.

### dCA measurement and data analysis

dCA measurements were performed in a quiet and temperature-controlled (22 °C–24 °C) room by the same professional technician from 14:30 to 17:30 with minimal sensory stimuli. Participants needed to pledge to refrain from high calorie meals for at least 4 h, moderate or more vigorous exercise for at least 12 h, and caffeine, nicotine, chocolate and alcohol intake for at least 12 h prior to examinations. In a supine position, participants had a 15-min period of rest with uncrossed legs before the assessment. TCD (Doppler Box, DWL, Germany) and servo-controlled finger photoplethysmography (Monitor 500 HD, CNAP, Austria) were simultaneously used to record continuous CBFV of the bilateral middle cerebral arteries and continuous BP values of the middle phalanx of the left middle finger, respectively. End-tidal carbon dioxide (EtCO_2_) was also recorded during the measurement using a capnograph (KMI605A, Kingst, China) through a nasal cannula. dCA was monitored for 30 min, including 10 min before acupuncture and 20 min after needle removal.

All the data were stored for offline analysis. As in previous studies (Zhang et al., 2022b; Zhang et al., 2022c), the analysis was performed based on the consensus white paper (Claassen et al., 2016) through MATLAB (MathWorks, United States) using scripts developed by the professional research team (Liu et al., 2020). Transfer function analysis (TFA) was applied to assess the dynamic relationship between CBFV and BP. Each participant’s CBFV and BP signals were aligned using a cross-correlation function. Then, the data were downsampled to 1 Hz after passing through an anti-aliasing third-order Butterworth low-pass filter with a cutoff frequency of 0.5 Hz. The averaged autospectra of ABP and CBFV, $\text{Sxx}\left(f\right)$ and $\text{Syy}\left(f\right)$ , and cross-spectra of ABP and CBFV, $\text{Sxy}\left(f\right)$ , were estimated using Welch’s method. The periodograms of downsampled ABP and CBFV were averaged in the frequency domain with a 50% overlapping Hamming window of 90 s. The transfer function, $H\left(f\right)$ , was then calculated as follows:

$$H\left(f\right)=\frac{\text{Sxy}\left(f\right)}{\text{Sxx}\left(f\right)}$$

Then, the phase shift, $\Phi \left(f\right)$ , and the gain, $\left|H\left(f\right)\right|$ , were calculated as follows from $H\left(f\right)$ :

$$\Phi \left(f\right)=\tan^{-1}\left[\frac{H_{I}\left(f\right)}{H_{R}\left(f\right)}\right]$$

$$\left|H\left(f\right)\right|=\sqrt{{\left|H_{I}\left(f\right)\right|}^{2}+{\left|H_{R}\left(f\right)\right|}^{2}}$$

where $H_{R}\left(f\right)$ and $H_{I}\left(f\right)$ denote the real and imaginary parts of the transfer function $H\left(f\right)$ , respectively. Then, the magnitude-squared coherence function was calculated as follows:

$$\gamma^{2}\left(f\right)=\frac{{\left|\text{Sxy}\left(f\right)\right|}^{2}}{\text{Sxx}\left(f\right)\text{Syy}\left(f\right)}$$

Phase, gain, and coherence were then derived from TFA to evaluate dCA. In general, the phase at low frequency (0.07–0.20 Hz) is the best for assessing the dCA. A higher phase in the low-frequency band indicates a better dCA. Because TFA is a linear model-based method, signals with low coherence between ABP and CBFV (according to the critical values of coherence provided in the CARNet white paper 2022 update (Panerai et al., 2022)) were excluded in the further statistical analysis.

### Statistical analysis

Statistical data were analyzed using SPSS Statistics version 17.0 (SPSS Inc., Chicago, IL, United States). Categorical variables were described as absolute values and percentages. The group differences for categorical variables were compared using the Pearson chi-square test or Fisher’s exact test. The normality of continuous data was tested by using the Shapiro–Wilk test. Normally distributed data were expressed as the mean ± standard deviation, while nonnormally distributed data were expressed as the median with interquartile range. The differences within the group for continuous variables were compared using the paired *t*-test or the Wilcoxon Signed-Ranks test. A value of *p* < 0.05 was considered statistically significant.

## Results

**FIGURE 3:**
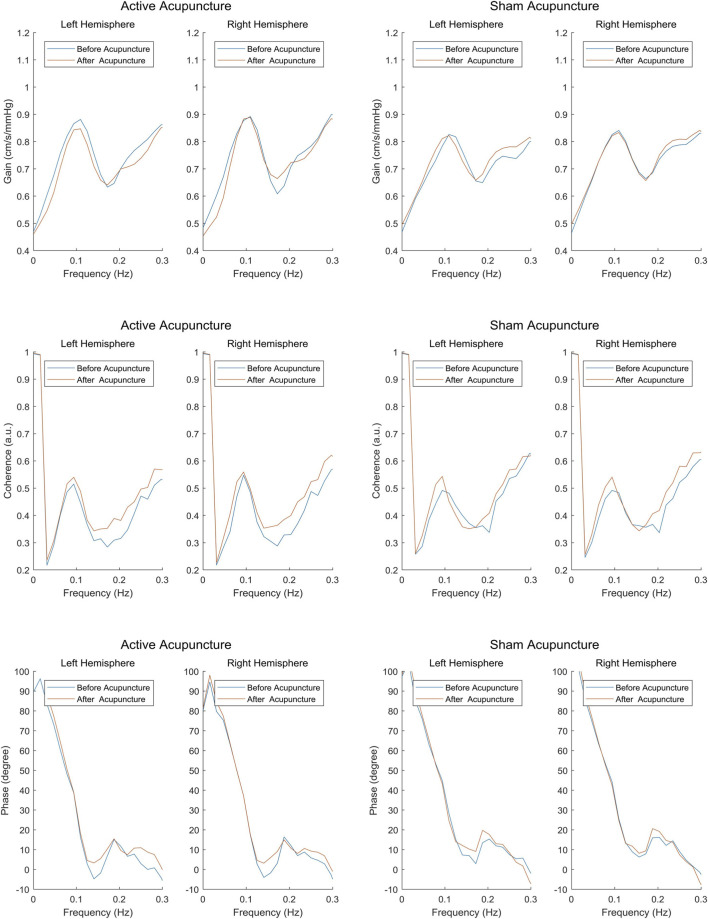
Changes in dCA parameters before and after acupuncture interventions.

Twenty healthy volunteers (25.3 ± 1.1 years; 10 male) were included in the study. As shown in Table 1, no significant differences in terms of age and body mass index were found between male and female. The changes in dCA parameters, CBFV, BP, EtCO_2_ and heart rate across either active or sham acupuncture interventions are presented in Table 2. There were no significant changes in mean MCA velocity, mean BP, and, EtCO_2_ after either active or sham acupuncture interventions. The left and right phase values in the low-frequency band after GB34 acupuncture treatment were significantly higher than baseline levels (Figure 3), while the other dCA parameters after GB34 acupuncture treatment were not significantly different from their baseline levels (Figure 3). In contrast, no real-time changes were observed in any of the dCA parameters after sham acupuncture intervention (Figure 3). There were significant real-time changes in heart rate after GB34 acupuncture treatment but no significant change in the sham acupuncture group.

**TABLE 1:** Demographic characteristics of participants.

Varibles	Male (n = 10)	Female (n = 10)	*P*
Age (y)	25.10 ± 0.99	25.50 ± 1.27	0.443
Body mass index	22.25 ± 2.46	20.09 ± 2.12	0.050

**TABLE 2:** dCA parameters, MCA velocity, mean BP, end-tidal CO_2_ and heart rate before and after intervention.

Parameter		Active acupuncture				Sham acupuncture			
		Before	After	*Z*	*P*	Before	After	*Z*	*P*
VLF (0.02–0.07 Hz) phase (degree)	Left	85.84 (68.76–103.10)	98.38 (78.92–109.83)	1.531	0.126	90.32 (77.95–99.09)	100.98 (81.85–113.08)	1.531	0.126
	Right	86.07 (63.64–97.22)	91.99 (79.31–106.01)	1.307	0.191	91.06 (75.86–101.32)	91.77 (76.86–106.06)	0.075	0.940
LF (0.07–0.20 Hz) phase (degree)	Left	24.88 (20.56–29.31)	29.03 (19.63–42.28)	2.203	**0.028**	28.98 (16.35–45.17)	31.12 (24.47–47.42)	1.120	0.263
	Right	23.50 (18.26–33.74)	29.15 (21.65–43.17)	2.389	**0.017**	29.78 (18.64–40.97)	30.35 (19.23–50.21)	0.560	0.575
HF (0.20–0.50 Hz) phase (degree)	Left	−3.47 (−9.77–5.43)	0.19 (−5.95–6.47)	0.635	0.526	0.46 (−4.15–5.74)	2.24 (−9.52–6.17)	0.224	0.823
	Right	−4.48 (−9.36–3.32)	−0.89 (−7.34–5.91)	0.560	0.575	−1.84 (−6.90–2.84)	−2.49 (−9.26–5.16)	1.045	0.296
VLF (0.02–0.07 Hz) gain (cm/s/mmHg)	Left	0.58 (0.48–0.66)	0.58 (0.50–0.59)	0.149	0.881	0.55 (0.47–0.65)	0.61 (0.49–0.65)	1.568	0.117
	Right	0.59 (0.49–0.65)	0.53 (0.47–0.62)	1.157	0.247	0.56 (0.48–0.62)	0.55 (0.49–0.66)	0.224	0.823
LF (0.07–0.20 Hz) gain (cm/s/mmHg)	Left	0.79 (0.67–0.84)	0.70 (0.66–0.79)	0.896	0.370	0.69 (0.64–0.82)	0.71 (0.65–0.80)	0.709	0.478
	Right	0.77 (0.68–0.84)	0.73 (0.66–0.83)	0.411	0.681	0.75 (0.64–0.79)	0.76 (0.70–0.81)	0.523	0.601
HF (0.20–0.50 Hz) gain (cm/s/mmHg)	Left	0.71 (0.63–0.86)	0.72 (0.65–0.79)	0.299	0.765	0.67 (0.61–0.77)	0.66 (0.60–0.84)	0.149	0.881
	Right	0.74 (0.63–0.85)	0.75 (0.69–0.82)	0.896	0.370	0.69 (0.61–0.82)	0.67 (0.61–0.84)	0.597	0.550
VLF (0.02–0.07 Hz) coherence	Left	0.60 (0.56–0.67)	0.62 (0.56–0.68)	0.299	0.765	0.60 (0.57–0.72)	0.62 (0.55–0.71)	0.037	0.970
	Right	0.60 (0.56–0.70)	0.59 (0.54–0.67)	1.008	0.313	0.60 (0.56–0.72)	0.64 (0.56–0.71)	0.112	0.911
LF (0.07–0.20 Hz) coherence	Left	0.32 (0.27–0.44)	0.30 (0.22–0.42)	0.075	0.940	0.35 (0.28–0.43)	0.27 (0.21–0.40)	1.568	0.117
	Right	0.34 (0.24–0.41)	0.30 (0.24–0.38)	0.261	0.794	0.34 (0.26–0.45)	0.31 (0.20–0.41)	1.269	0.204
HF (0.20–0.50 Hz) coherence	Left	0.21 (0.12–0.31)	0.22 (0.16–0.33)	0.672	0.502	0.28 (0.19–0.38)	0.23 (0.18–0.43)	0.821	0.411
	Right	0.23 (0.14–0.34)	0.24 (0.16–0.35)	1.157	0.247	0.28 (0.14–0.38)	0.24 (0.18–0.39)	0.299	0.765
MCA velocity (cm/s)	Left	75.16 (62.77–80.31)	72.32 (59.57–79.76)	1.381	0.167	74.74 (65.94–85.47)	73.28 (65.91–84.59)	0.485	0.627
	Right	76.10 (62.17–89.60)	73.94 (64.74–86.19)	0.747	0.455	71.45 (64.11–84.41)	69.87 (65.04–81.17)	0.112	0.911
Mean BP (mmHg)		73.65 (67.91–77.57)	75.69 (68.15–81.02)	1.941	0.052	74.60 (65.07–79.12)	74.91 (70.29–84.86)	1.381	0.167
End-tidal CO_2_ (mmHg)		28.36 (26.09–32.87)	29.10 (25.08–31.69)	1.904	0.057	28.75 (23.68–33.00)	28.42 (21.86–31.92)	1.269	0.204
Heart rate (bpm)		73.58 (68.90–78.35)	70.81 (66.62–74.20)	2.427	**0.015**	74.55 (66.13–80.34)	74.68 (62.22–77.71)	1.829	0.067

The meaning of the bold values: *p* < .05.

## Discussion

This first study to investigate real-time changes in dCA after acupuncture showed that dCA improved after active acupuncture at GB34 in healthy adults but did not improve after sham acupuncture. Meanwhile, acupuncture at GB34 was also associated with a change in heart rate.

Several studies have indicated that GB34 is beneficial for the improvement of motor function. Acupuncture stimulation at GB34 modulates the cortical activities of the somatomotor area (Jeun et al., 2005). Acupuncture at GB34 could enhance motor recovery by strengthening causal influences between the ipsilesional and contralesional motor cortex (Bai et al., 2013). A resting-state fMRI study showed that acupuncture at GB34 could increase the intrinsically decreased functional connectivity between the bilateral primary motor cortices to recover motor function in stroke patients (Ning et al., 2017). Another resting-state fMRI study demonstrated that acupuncture at GB34 could activate all levels of brain networks, including cognitive, motor, default network, limbic system and other parts of the encephalic region (Liu et al., 2018). Based on this, acupuncture at GB34 could promote the rehabilitation of hemiplegic patients by increasing motor-cognition connectivity (Chen et al., 2015). In addition, acupuncture at GB34 can enhance cerebral blood flow and ameliorated spastic paresis by up-regulating the expressions of GABAAγ2 and KCC2 in the ischemic cortex as well as lumber enlargement (Zhou et al., 2023). In the present study, the bilateral phase was increased after acupuncture with GB34, providing additional objective evidence for the therapeutic effect of GB34.

Although the mechanism by which acupuncture at GB34 improves dCA remains unclear, we explored a discussion of theoretical possibilities. CA is defined as the ability to maintain stable CBF in the process of BP changes, realized through myogenic, neurogenic, endothelial, and metabolic mechanisms (Paulson et al., 1990). Cortical microvessels can be regulated by cells located in subcortical areas and within the cerebral cortex through vasoactive mediators, such as nitric oxide (NO), neuropeptide Y, and serotonin (Hamel, 1985). A previous study showed that acupuncture plays a role in reducing oxidative stress and increasing NO bioavailability and endothelial function (Leung et al., 2016). Acupuncture can influence the NO level and increase the local circulation (Tsuchiya et al., 2007). Acute acupuncture treatment is helpful for improving endothelial dysfunction (Park et al., 2010). Acupuncture also induces neural plasticity, which is likely associated with its modulation of neurotrophins and neurotransmitters (Xiao et al., 2018). According to research, acupuncture can protect neurons via mobilizing endogenous protective mechanisms within the brain (Fu et al., 2024). Therefore, it is possible that the improvement in dCA after acupuncture with GB34 is achieved through these mechanisms.

Previous studies on the effect of acupuncture on CBF regulation have mainly focused on cerebrovascular reactivity. GV14 acupuncture treats disorders of anterior and posterior cerebral circulation by increasing CO_2_ reactivity in the MCA and basilar artery (Lee et al., 2021). GB20 acupuncture can treat disorders of posterior cerebral circulation by increasing CO_2_ reactivity in the basilar artery but has no effect on the MCA (Im et al., 2014). GB34 acupuncture treatment improved CO_2_ reactivity, specifically in the ipsilateral MCA, but had no effect on either the anterior cerebral artery (ACA) or the contralateral MCA (Moon et al., 2019), which is both different and related to the results of our study. The above results suggested that acupuncture is effective in improving local CBF. CBF regulation plays an important role in the occurrence, development and prognosis of diseases (Liu et al., 2022). Therefore, our study suggests that GB34 acupuncture treatment may contribute to cerebrovascular diseases, other nervous system diseases, systemic diseases, and psychiatric diseases.

The accumulated evidence has proven that CA is significantly associated with the severity and outcome of diseases and suggests that targeting improvement of CA should be a potential coadjutant in future therapy (Claassen et al., 2021). However, at present, only a few interventions have been proven to improve dCA. A phase II randomized placebo-controlled trial observed that acute treatment with pravastatin after aneurysmal subarachnoid hemorrhage improves dCA (Tseng et al., 2005). In a study comparing the effects of midazolam and propofol sedation on dCA, only midazolam sedation was likely to improve dCA in healthy subjects (Ogawa et al., 2010). Deferoxamine may improve dCA in healthy volunteers, especially in elderly patients, by activating hypoxia-inducible transcription factor-1 (Sorond et al., 2015). It should be noted that the utility of the three drugs for these effects have not yet been verified by large-scale clinical trials. Therefore, they should not be used beyond the indications in clinics. Recently, a randomized, controlled, multicenter trial showed that butylphthalide treatment for 90 days can significantly improve dCA in patients with acute ischemic stroke (Guo et al., 2023). In addition, the effects of nondrug interventions on dCA are worthy of further exploration. External counterpulsation enhanced cerebral perfusion for at least 3 weeks in patients with ischemic stroke (Xiong et al., 2016). Another novel treatment, remote ischemic conditioning (RIC), induced a sustained increase in dCA after treatment in healthy adults (Guo et al., 2019; Qu et al., 2022). Our study revealed a simple and economical TCM therapy that can improve dCA rapidly and has beneficial effects on cerebrovascular function.

The heart rate decreased after acupuncture at GB34, and this was considered to be related to heart rate variability (HRV). A previous exploratory and preliminary study suggested that acupuncture could affect cardiac autonomic neural regulation via the parasympathetic system, manifesting as a reduction in heart rate and increased HRV (Huang et al., 2015). Acupuncture improved cerebral hemodynamics by increasing parasympathetic tone to activate the autonomic nervous system (Hyun et al., 2014). Therefore, there is a potential association between HRV and dCA (Quispe-Cornejo et al., 2023; Mankoo et al., 2023).

In recent years, fMRI and EEG have been widely to measure the modulation effect of acupuncture on brain activity and cognition with brain-computer interface. A fMRI regional homogeneity-based meta-analysis showed the precise brain region response targets of acupuncture for mild cognitive impairment (Ma et al., 2022). The therapeutic effect of acupuncture on human brain can be evaluated by extracting periodic-aperiodic features (Yu et al., 2024), functional connectivity (Yu et al., 2018), and power changes (Rao et al., 2025) of EEG. In addition, the EEG decoder provides an acupuncture-brain interface linking somatosensory stimulations with neural representations, an effective scheme for revealing clinical efficacy of acupuncture treatment (Yu et al., 2025). Nowadays, multimodal monitoring is the current trend. The good spatial resolution of fMRI and EEG combined with the temporal resolution of dCA may reflect the complex cooperation of vascular neural network. In the future, it is possible to combine spatio-temporal features of brain activity and dCA to evaluate clinical effect of acupuncture.

There are some limitations to this study. First, we only observed the transient change in dCA after acupuncture and did not observe how long the effect lasted. Further research into the long-term efficacy of acupuncture with regard to dCA is needed. Second, the participants were young healthy volunteers and the sample size was relatively small. With the results from our study, we can safely conclude only that the present conclusion is applicable to healthy young adults without the conditions described in the Methods. It is unknown whether effects will be generated as quickly in other age groups. It is still unclear whether acupuncture can also improve dCA in patients with various cerebrovascular and neurologic diseases such as ischemic stroke. Finally, only hemodynamic parameters were involved in this study. It is worth studying whether the biomarkers related to dCA change during acupuncture.

## Conclusion

Overall, the main finding is an improvement in dCA indices (LF phase) in young healthy adults after acupuncture at GB34. Our study provides objective evidence of acupuncture and a new approach to improve the cerebrovascular function in terms of dCA.

## Data Availability

The raw data supporting the conclusions of this article will be made available by the authors, without undue reservation.
